# Effect of melatonin for the management of endometriosis: A protocol of systematic review and meta-analysis

**DOI:** 10.1097/MD.0000000000020353

**Published:** 2020-05-29

**Authors:** Ping Chen, Dong-xu Zhao, Lei Chen, Cui-hong Su, Yan-jia Ji, Dong-wei Wang

**Affiliations:** a Department of Obstetrics and Gynecology, First Affiliated Hospital of Jiamusi University, Jiamusi, 154002, China; b Department of Obstetrics and Gynecology, Jiamusi Central Hospital, Jiamusi, 154002, China; c Department of Anesthesiology, First Affiliated Hospital of Jiamusi University, Jiamusi, 154002, China.

**Keywords:** effect, endometriosis, melatonin

## Abstract

**Background:**

This study aims to explore the effect of melatonin for the management of endometriosis.

**Methods:**

We will search electronic databases (Cochrane Library, MEDLINE, EMBASE, CINAHL, Web of Science, Scopus, Allied and Complementary Medicine Database, Chinese Biomedical Literature Database, and China National Knowledge Infrastructure) from their inceptions to the February 29, 2020 without language and publication time limitations. The study identification, study quality assessment, and data extraction will be undertaken by two separate researchers. We will also appraise evidence quality of main outcomes by Grading of Recommendations Assessment Development and Evaluation, and statistical analysis performance by RevMan 5.3 Software.

**Results:**

This study will summarize up-to-date clinical evidence to investigate the effect of melatonin for the management of endometriosis.

**Conclusion:**

This study may provide helpful evidence of melatonin for the management of endometriosis.

**Systematic review registration:**

INPLASY202040093.

## 1 Introduction

Endometriosis is a common estrogen-dependent inflammatory disorder in females,^[1–3]^ which often manifests as debilitating chronic pelvic pain, infertility, and fatigue.^[4–7]^ It has been estimated that this condition can affect 6–10% females of reproductive age, and more than 50% of infertile females.^[8–9]^ If patients with such condition can not be treated fairly well, it can result in very poor quality of life in those patients.^[10–13]^

During the past decades, despite a numerous therapies have been developed to manage patients with endometriosis, but all their efficacy is still not satisfied and also accompany with a variety of adverse events.^[11,14–18]^ Thus, exploring new medication is still very urgent. Several studies reported that melatonin can help manage endometriosis.^[19–22]^ However, no study addresses this topic with higher level evidence systematically. Therefore, the target of this study is to assess the effect of melatonin for the management of endometriosis.

## 2 Methods and analysis

### 2.1 Study registration

This study was registered through INPLASY202040093. Its report follows the guideline of Preferred Reporting Items for Systematic Reviews and Meta-Analysis Protocol statement.^[23]^

### 2.2 Dissemination and ethics

We will publish this study on a peer-reviewed journal or a relevant conference. This study is a literature research, thus, ethic approval is not needed.

### 2.3 Eligibility criteria

#### 2.3.1 Participants/subjects

This study will include patients who were diagnosed as endometriosis or normal participants without restrictions to the race, age, and gender.

#### 2.3.2 Interventions/exposure

All studies utilized melatonin alone will be included in the intervention group.

All studies used any other management, except melatonin will be included in the control group.

#### 2.3.3 Study types

We will include randomized controlled trials that investigated the effect of melatonin for the management of endometriosis. No language and publication time limitations will be applied to this study.

#### 2.3.4 Outcome measurements

Primary outcomes are endometriosis cytokines (including Bcl-2, Fas, ICAM-1), as measured by immunohistochemistry; and natural killer cell activity, as detected by MTT Assay Kit.

Secondary outcomes are serum levels of Tumor Necrosis Factor-α, Interleukin-6, and Interleukin IL-8.

### 2.4 Literature search

Electronic databases (Cochrane Library, MEDLINE, EMBASE, CINAHL, Web of Science, Scopus, Allied and Complementary Medicine Database, Chinese Biomedical Literature Database, and China National Knowledge Infrastructure) will be searched from their inceptions until February 29, 2020 with no language and publication time restrictions. The search strategy for Cochrane Library is presented (Table 1). We will modify equivalent search strategies for other electronic databases.

**Table 1:**
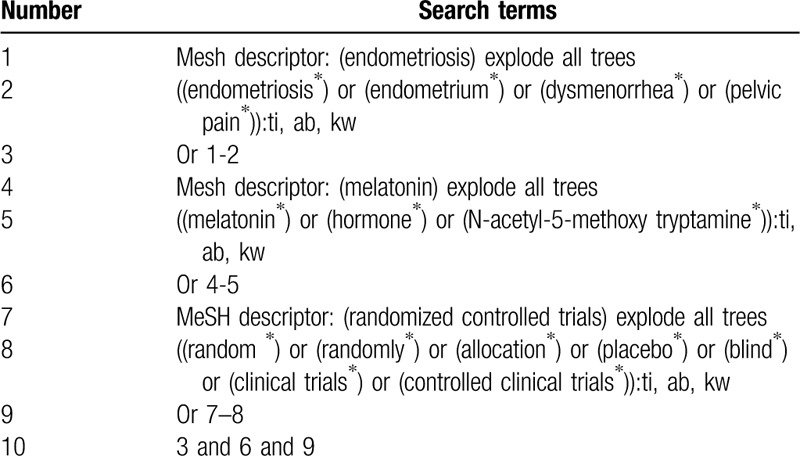
Search strategy applied in Cochrane Library.

This study will search other literature sources, such as Google Scholar, conference abstract, and reference lists of all included studies.

### 2.5 Literature selection

All searched studies will be managed with NoteExpress 3.2.0, and we will exclude duplicates. Two separate researchers will examine titles/abstracts of all potential studies based on all eligibility criteria. Then, full-texts of all remaining articles will be further identified to make the final decision and all eligible articles will be included. During the procedure of study selection, divergences will be solved by discussion with consultation from a third researcher. We will summarize the results of study selection in a flowchart.

### 2.6 Data extraction and management

Two researchers will separately extract the following information: title, first author, year of publication, patient characteristics, study methods, study setting, details of intervention and controls (e.g. types of delivery, dosage, and frequency), outcomes, adverse events, results, findings, and funding information. Any conflicts between two researchers will be cleared up by discussion with a third researcher. We will contact original authors to request any missing or unclear data, and we will use intention-to-treat analysis for data analysis if we can not achieve that data.

### 2.7 Risk of bias assessment

Risk of bias for each included study will be appraised by two independent researchers through Cochrane risk of bias tool. It consists of 7 domains, and each one is graded as low, unclear, or high risk of bias. If any different views will be identified between both of them, we will invite a third researcher to solve them through discussion.

### 2.8 Statistical analysis

#### 2.8.1 Data synthesis

This study will use RevMan 5.3 software to perform statistical analysis. We will estimate continuous data as weighted mean difference or standardized mean difference and 95% confidence intervals, and dichotomous data as risk ratio and 95% confidence intervals. We will identify statistical heterogeneity by *I*^*2*^ test. In accordance with the statistical heterogeneity levels among eligible studies, a fixed-effects model (*I*^*2*^ ≤50%) or a random-effects model (*I*^*2*^ >50%) will be applied to pool the extracted outcome data. If *I*^*2*^ ≤50%, we will carry out a meta-analysis. Otherwise, if *I*^*2*^ >50%, we will perform a subgroup analysis or sensitivity analysis to find possible sources from clinical and methodological aspects.

#### 2.8.2 Subgroup analysis

If necessary, we will carry out a subgroup analysis based on the study characteristics, details of treatment and controls, and outcomes.

#### 2.8.3 Sensitivity analysis

Whenever necessary, we will also conduct a sensitivity analysis to test stability of results according to the sample size, and study quality.

#### 2.8.4 Reporting bias

When sufficient included studies are included, we will plan to undertake a funnel plot and Egger's regression test to find out reporting bias.^[24]^

#### 2.8.5 Grading the quality of evidence

Quality of evidence for all major outcomes will be assessed by two separate researchers using Grading of Recommendations Assessment Development and Evaluation.^[25]^ If we find out any divisions, we will consult a third researcher for help and a final decision will be reached.

## 3 Discussion

To our best knowledge, no previous systematic review has explored the effect of melatonin for the management of endometriosis. This study will firstly evaluate the effect of melatonin for endometriosis. We will identify as comprehensive literature sources as possible without language limitations. All potential studies regarding the effect of melatonin for the management of endometriosis will be fully considered. The findings of this study may present an up-to-date summary of the latest evidence on the effect of melatonin for endometriosis.

## Acknowledgments

This work has been supported by the Heilongjiang Provincial Health and Health Committee General Program (2018–329), and Jiamusi University Teaching and Research General Program (2016JY1035, 2017YYL-048). The financial supporter will not participate any parts of this study.

## Author contributions

**Conceptualization:** Ping Chen, Dong-xu Zhao, Lei Chen, Cui-hong Su, Dong-wei Wang.

**Data curation:** Yan-jia Ji, Dong-wei Wang.

**Formal analysis:** Ping Chen, Lei Chen, Cui-hong Su, Yan-jia Ji, Dong-wei Wang.

**Investigation:** Lei Chen, Dong-wei Wang.

**Methodology:** Ping Chen, Dong-xu Zhao, Lei Chen, Cui-hong Su, Yan-jia Ji.

**Project administration:** Dong-wei Wang.

**Resources:** Ping Chen, Dong-xu Zhao, Lei Chen, Cui-hong Su, Yan-jia Ji.

**Software:** Ping Chen, Dong-xu Zhao, Lei Chen, Cui-hong Su, Yan-jia Ji.

**Supervision:** Dong-wei Wang.

**Validation:** Ping Chen, Dong-xu Zhao, Cui-hong Su.

**Visualization:** Ping Chen, Lei Chen, Cui-hong Su, Yan-jia Ji, Dong-wei Wang.

**Writing – original draft:** Ping Chen, Dong-xu Zhao, Yan-jia Ji, Dong-wei Wang.

**Writing – review & editing:** Ping Chen, Lei Chen, Cui-hong Su, Yan-jia Ji, Dong-wei Wang.
